# How efficient is patient discharge following lower limb arthroplasty?

**DOI:** 10.1186/s13741-015-0015-y

**Published:** 2015-04-30

**Authors:** Elizabeth Ashby, Claire Matejowsky, Michael G Mythen, Fares S Haddad, Michael PW Grocott

**Affiliations:** Catterall Unit, Royal National Orthopaedic Hospital, Brockley Hill, Stanmore, Middlesex HA7 4LP UK; Surgical Outcomes Research Centre, University College London Hospital, 235 Euston Road, Fitzrovia, London, NW1 2BU UK; UCL Institute of Child Health, Surgical Outcomes Research Centre, Joint UCLH/UCL Comprehensive Biomedical Research Centre, Gower Street, London, WC1E 6BT UK; University College London Hospital, 235 Euston Road, Fitzrovia, London, NW1 2BU UK; Integrative Physiology and Critical Illness Group, Clinical and Experimental Sciences, University of Southampton, University Road, Southampton, SO17 1BJ UK; NIHR Respiratory Biomedical Research Unit, University Hospital Southampton/University of Southampton, University Road, Southampton, SO17 1BJ UK

**Keywords:** Bed utilisation, Arthroplasty, Post-operative morbidity

## Abstract

**Background:**

Appropriately timed patient discharge is essential for optimal patient care and efficient hospital functioning. The post-operative morbidity survey (POMS) is the only validated prospective method of measuring short-term post-operative morbidity. It has not previously been used as a bed utilisation tool.

**Methods:**

We collected POMS data from 529 consecutive lower-limb arthroplasty patients over a 1-year period and recorded the number of patients remaining in the hospital without morbidity, together with alternative reasons for remaining in hospital. Data was collected on post-operative days (POD) 3, 5, 8 and 15.

**Results:**

On POD 3, 45% of hip arthroplasty patients and 52% of knee arthroplasty patients remained in hospital with no identifiable morbidity. On POD 5, 53% of hip arthroplasty patients and 47% of knee arthroplasty patients remained in hospital with no identifiable morbidity. These figures declined by POD 8 and 15. The most common reason for inappropriate bed occupancy was ongoing physiotherapy and occupational therapy.

**Conclusions:**

We believe POMS is able to identify patients remaining in hospital with no significant morbidity and has utility as a prospective bed utilisation tool. Addition of a mobility measure to POMS may improve its utility in detecting morbidity requiring hospitalisation, particularly following lower limb arthroplasty.

## Background

Appropriately timed discharge of patients following surgery is essential for optimal patient care and efficient hospital functioning. A patient discharged early is at risk of under-diagnosis of medical complications with consequent adverse outcome. A patient whose discharge is delayed is at risk of developing a hospital-associated complication (for example, hospital-acquired infection) and incurs an unnecessary cost to the health-care provider. Post-operative patients should be discharged at the earliest safe opportunity to reduce the rate of hospital-associated complications and the cost of each in-patient episode. Appropriate discharge timing should increase the throughput of patients and reduce waiting times.

Historically, hospitals in the UK have been paid according to contracts with no financial incentive to treat increased numbers of patients. This changed in 2000 when the National Health Service (NHS) Plan [1] announced that hospital incomes would be directly linked to activity. Payment by results [2] began in 2003 and now every healthcare provider is paid a sum (tariff) for each procedure undertaken. In the UK, many patients remain in hospitals with no medical indication [3]. One study showed that 31% of post-operative patients remained in hospitals inappropriately [4]. Payment by results aims to reduce this figure by rewarding efficiency and encouraging increased activity.

In order to improve efficiency, hospitals must first recognise inappropriate bed occupancy. The post-operative morbidity survey (POMS) [5] is the only validated prospective method of measuring short-term post-operative morbidity in the literature. POMS was designed to identify morbidity of a type and severity that could delay hospital discharge. The survey focuses on indicators of organ system dysfunction (for example, inability to tolerate enteral diet) rather than traditional diagnostic categories (for example, deep vein thrombosis). POMS assesses nine domains of morbidity (Table 1). Data is obtained from observation charts, medication charts, patient notes, routine blood test results and direct patient questioning and observation. No additional investigations are required. The data collection process is simple to allow routine screening of large numbers of patients. POMS has been shown to be reliable, valid and acceptable to patients [6]. POMS has been used in outcomes research [7] and in effectiveness research [8].

**Table 1:** **Criteria for a positive POMS score**

**Variable**	**Criteria for positive result**
Pulmonary	Requires supplementary oxygen or ventilatory support
Infection	Currently on antibiotics or temperature >38°C in the last 24 hours
Renal	Oliguria (<500 ml/day), elevated creatinine (>30% pre-op level), catheter *in situ* (for non-surgical reason)
Gastrointestinal	Unable to tolerate enteral diet for any reason
Cardiovascular	Diagnostic tests or treatment within the last 24 h for: myocardial infarction, hypotension (requiring pharmacological therapy or fluids >200 ml/h), atrial/ventricular arrhythmia or cardiogenic pulmonary oedema
Central nervous system	Presence of new focal deficit, coma, confusion or delirium
Wound complications	Wound dehiscence requiring surgical exploration or drainage of pus from operative wound with or without isolation of organisms
Haematological	Requirement of blood transfusion, platelets, fresh frozen plasma, or cryoprecipitate within the last 24 h
Pain	Wound pain requiring parenteral opioids or regional anaesthesia

In the US, over 98% of post-operative in-patients had morbidity defined by POMS [5]. This implies that patients with a POMS score of 0 are fit for discharge.

Therefore, as well as providing useful clinical research and audit data, POMS may have utility for assessing and improving hospital bed utilisation.

The aim of this study is to identify inappropriate bed occupancy following lower limb arthroplasty using POMS. We report the reasons for delayed discharge and suggest ways to improve bed utilisation.

## Methods

Ethical approval was gained from the Joint UCLH/UCL Committee on the Ethics of Human Research (ref. number 01/0116) prior to commencement of the study. The requirement for consent was waived as collection of POMS has become a routine part of service evaluation within our institution. All patients aged 18 or over undergoing elective lower limb arthroplasty at University College Hospitals NHS Trust over a 12-month period were eligible for inclusion into this prospective cohort study. There were no exclusion criteria ensuring a consecutive sample was taken. Elective lower limb arthroplasty procedures included unicompartmental knee replacement (UKR), total knee replacement (TKR), revision total knee replacement, hip resurfacing (HR), total hip replacement (THR) and revision total hip replacement.

Data was collected by one of two study nurses. The age, sex, American Society of Anesthesiologists (ASA) physical status score and length of in-patient stay for each patient were recorded. POMS data were collected on post-operative days (POD) 3, 5, 8 and 15 if the patient remained in hospital. Presence of morbidity was defined as occurring in any patient meeting POMS criteria in one or more domains of the survey on the day of data collection. For patients remaining in hospital without morbidity on POD 8 and POD 15, the reason was recorded. The use of mobility aids on these days was also noted.

The number and percentage of patients with no identifiable morbidity according to POMS was calculated for POD 3, 5, 8 and 15. The number of days each patient remained in hospital with no morbidity was calculated by distracting the day on which the patient first had a POMS score of 0 from his or her total length of stay. An overall estimated cost saving was calculated by multiplying this figure by the average cost for one orthopaedic in-patient night.

The number of patients developing postoperative morbidity after a period free of morbidity was recorded. The number of readmissions to the same hospital in the first year following discharge was also recorded.

## Results

Data collection was completed on 529 patients. Patient characteristics of the study population are shown in Table 2. The mean age of all study patients was 68.9 years, the median ASA physical status score was 2 and 62% of patients were female. The median length of stay was 7 days, and the overall in-patient mortality rate was 0.4%.

**Table 2:** **Demographics of the study population**

	**Number performed**	**Mortality rate (%)**	**Age (years)**		**ASA physical status score**		**% Male**	**Length of stay (days)**	
			**Mean**	**Range**	**Median**	**Range**		**Median**	**Range**
UKR	66	1	66.1	45 to 87	2	1 to 3	45	5	2 to 52
TKR	226	0	70.3	23 to 90	2	1 to 3	36	6	3 to 37
RTKR	8	0	71.6	46 to 88	2	1 to 3	25	13	3 to 102
HR	32	0	51.6	22 to 70	1	1 to 3	50	6	4 to 13
THR	162	0	70.7	21 to 89	2	1 to 3	36	8	3 to 51
RTHR	35	3	72.2	26 to 88	2	1 to 3	36	14	6 to 93
Total	529	0.4	68.9	21 to 90	2	1 to 3	38	7	2 to 102

### A) Hip arthroplasty patients

The location of hip arthroplasty patients on POD 3, 5, 8 and 15 is shown in Table 3. Many patients undergoing HR remained in the hospital with no identified morbidity on POD 3 (75%), 5 (78%) and 8 (16%). All HR patients had been discharged by POD 15.

**Table 3:** **Location of patients following hip arthroplasty**

			**POD 3**	**POD 5**	**POD 8**	**POD 15**
Procedure	HR	Patients discharged	0/32 (0%)	2/32 (6%)	27/32 (84%)	32/32 (100%)
		Inpatients POMS >0	8/32 (25%)	5/32 (16%)	0/32 (0%)	0/32 (0%)
		Inpatients POMS = 0	24/32 (75%)	25/32 (78%)	5/32 (16%)	0/32 (0%)
	THR	Patients discharged	0/162 (0%)	13/162 (8%)	78/162 (48%)	138/162 (85%)
		Inpatients POMS >0	87/162 (54%)	62/162 (38%)	29/162 (18%)	13/162 (8%)
		Inpatients POMS = 0	75/162 (46%)	87/162 (54%)	55/162 (34%)	11/162 (7%)
	Revision THR	Patients discharged	0/35 (0%)	0/35 (0%)	3/35 (9%)	20/35 (57%)
		Inpatients POMS >0	31/35 (89%)	25/35 (71%)	21/35 (60%)	14/35 (40%)
		Inpatients POMS = 0	4/35 (11%)	10/35 (29%)	11/35 (31%)	1/35 (3%)
	TOTAL	Patients discharged	0/230 (0%)	16/230 (7%)	109/230 (47%)	191/230 (83%)
		Inpatients POMS >0	127/230 (55%)	92/230 (40%)	50/230 (22%)	27/230 (12%)
		Inpatients POMS = 0	103/230 (45%)	122/230 (53%)	71/230 (31%)	12/230 (5%)

**Figure 1:**
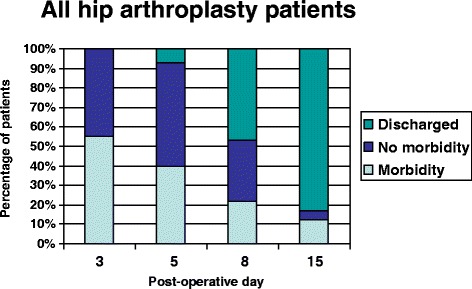
Discharge status and prevalence of morbidity following all types of hip arthroplasty.

Many THR patients remained in hospital with no identifiable morbidity on POD 3 (46%), 5 (54%), 8(34%) and 15 (7%). Patients undergoing revision THR patients remained in the hospital with no identifiable morbidity on POD 3 (11%), 5 (29%), 8 (31%) and 15 (3%). Discharge status and prevalence of morbidity for all hip arthroplasty patients combined are presented in Figure 1.

### B) Knee arthroplasty patients

The location of knee arthroplasty patients on POD 3, 5, 8 and 15 is shown in Table 4. Many patients undergoing UKR remained in the hospital with no identified morbidity on POD 3 (63%), 5 (39%) and 8 (6%). All UKR patients had been discharged by POD 15.

**Table 4:** **Location of patients following knee arthroplasty**

			**Day 3 post-op**	**Day 5 post-op**	**Day 8 post-op**	**Day 15 post-op**
Procedure	UKR	Patients discharged	7/66 (11%)	33/66 (50%)	59/66 (89%)	65/66 (98%)
		Inpatients POMS >0	17/66 (26%)	7/66 (11%)	3/66 (5%)	1/66 (2%)
		Inpatients POMS = 0	42/66 (63%)	26/66 (39%)	4/66 (6%)	0/66 (0%)
	TKR	Patients discharged	0/226 (0%)	22/226 (10%)	145/226 (64%)	211/226 (93%)
		Inpatients POMS >0	114/226 (50%)	90/226 (40%)	38/226 (17%)	7/22 (3%)
		Inpatients POMS = 0	112/226 (50%)	114/226 (50%)	43/226 (19%)	8/226 (4%)
	Revision TKR	Patients discharged	0/8 (0%)	1/8 (13%)	1/8 (13%)	6/8 (75%)
		Inpatients POMS >0	6/8 (75%)	4/8 (50%)	5/8 (62%)	1/8 (12.5%)
		Inpatients POMS = 0	2/8 (25%)	3/8 (37%)	2/8 (25%)	1/8 (12.5%)
	TOTAL	Patients discharged	7/300 (2%)	56/300 (19%)	205/300 (68%)	282/300 (94%)
		Inpatients POMS >0	137/300 (46%)	101/300 (34%)	46/300 (15%)	9/300 (3%)
		Inpatients POMS = 0	156/300 (52%)	143/300 (47%)	49/300 (17%)	9/300 (3%)

**Figure 2:**
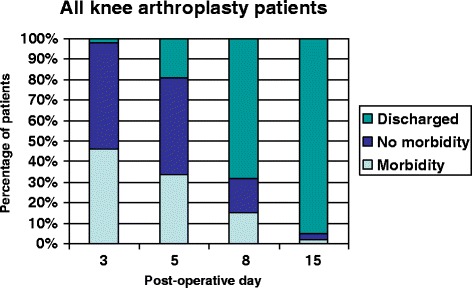
Discharge status and prevalence of morbidity following all types of knee arthroplasty.

Many TKR patients remained in the hospital with no identifiable morbidity on POD 3 (50%), 5 (50%), 8 (19%) and 15 (4%). Revision TKR patients remained in the hospital with no identifiable morbidity on POD 3 (52%), 5 (47%), 8 (17%) and 15 (3%). Discharge status and prevalence of morbidity for all knee arthroplasty patients combined are presented in Figure 2.

### C) Overall inappropriate bed occupancy days

Table 5 shows the average number of days that post-operative patients remained in the hospital with no identifiable morbidity. HR patients stayed an average of 2.36 days with no morbidity, THR patients 4.19 days and revision THR patients 10.37 days. UKR patients stayed an average of 1.76 days with no identifiable morbidity, TKR patients 2.73 days and revision TKR patients 14.38 days.

**Table 5:** **Number of inappropriate inpatient days classified by type of arthroplasty**

	**Total number of patients**	**Total number of inappropriate in-patient days**	**Average number of inappropriate in-patient days per patient**
HR	33	78	2.36
THR	162	678	4.19
Revision THR	35	363	10.37
UKR	63	111	1.76
TKR	227	620	2.73
Revision TKR	8	115	14.38
Total	528	1965	3.72

### D) Cost of inappropriate bed occupancy days

529 patients were included in this study. These patients remained in the hospital for a total of 1,965 days with no morbidity as defined by POMS. A surgical in-patient bed costs up to £400 per night [9]. If these patients had been discharged when their POMS score was 0, a saving of up to £786,000 could have been made.

### E) Reasons for patients with no morbidity remaining in hospital

**Figure 3:**
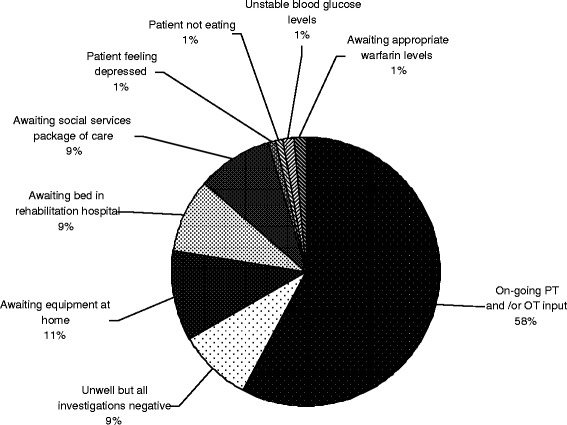
Reasons lower-limb arthroplasty patients with no morbidity remained in the hospital on post-operative days 8 and 15.

Of the 529 patients participating in this study, 120 remained in hospital with no morbidity defined by POMS on POD 8, and 20 patients remained with no identifiable morbidity on POD 15. The reasons for non-discharge are shown in Figure 3. The most common reason is continuing physiotherapy and occupational therapy input. Other reasons include waiting for home equipment, waiting for a rehabilitation bed, waiting for a social services package of care and patients feeling unwell with negative investigations.

Of the patients remaining in hospital with no morbidity identified by POMS, 24% were mobilising with a zimmer frame, 55% were mobilising with two crutches, 14% with a single crutch and 7% were mobilising unaided. This study did not record how far patients could mobilise or whether they could climb stairs.

### F) New morbidity and readmission

Thirty-eight out of 529 patients developed morbidity as an in-patient following a period without morbidity. Five of these patients underwent a second surgical procedure and developed morbidity in the second post-operative period. Thirty patients (6.2%) developed morbidity following a period without morbidity. Twenty-five of these patients developed wound morbidity: 24 patients had no morbidity on POD 3 but developed wound morbidity by POD 5, and 1 revision arthroplasty patient had no morbidity on POD 5 but developed wound morbidity by POD 8. Six patients developed cardiovascular morbidity after a period with no morbidity: 5 patients were prescribed anticoagulation (2 for pulmonary embolus and 3 for deep vein thrombosis) and 1 patient had a myocardial infarction. One patient developed neurological morbidity (a cerebro-vascular accident) after a period without morbidity, and 1 patient developed infectious morbidity (an infected peripheral cannula site) after a period without morbidity.

No patient in this study was readmitted to the same hospital in the first year following discharge for any reason relating to their surgery.

## Discussion

This study identifies many patients remaining in hospital with no identifiable morbidity following lower-limb arthroplasty in a UK teaching hospital. The rate of inappropriate bed occupancy varies with time after surgery and type of arthroplasty.

The most common reason for patients remaining in hospital with no identifiable morbidity was ongoing physiotherapy and occupational therapy. This suggests that improving pre- and post-operative planning could improve appropriate bed occupancy. Patients could be taught post-operative physiotherapy exercises in group classes prior to surgery. Occupational therapists could assess each patient’s home environment and ensure necessary modifications are made. In the post-operative period ‘fast-track’ pathways could be used to ensure maximum therapy input at the earliest possible opportunity. Some physiotherapy and occupational therapy could be provided post-operatively in a rehabilitation unit or at the patient’s home rather than in hospital. This would require safety and cost evaluation prior to implementation.

Three of the top five reasons for patients remaining in hospital with no identifiable morbidity relate to ‘social’ issues (awaiting home equipment, awaiting a rehabilitation bed, awaiting a package of care from social services). Pre-operative clinics could identify and address these problems prior to admission. Such clinics could also be used to manage patient expectation so they are aware of the difficulties they may encounter in the post-operative period and the expected timing of discharge.

This study has several strengths. A large consecutive dataset was collected prospectively using a validated methodology for measuring post-operative morbidity. This is the first published study to prospectively evaluate the appropriateness of discharge following lower limb joint replacement surgery.

The weaknesses of this study are that it was conducted in a single centre, POMS is not validated as a bed utilisation tool, there was not daily recording of data so the calculation of excess days are an approximation, and patient mobility was not fully assessed. Data was collected regarding mobility aids, but the distance each patient could mobilise was not recorded.

This is the first time that POMS has been used as a bed utilisation tool. It has not been validated for this purpose but has previously been used to identify patients in hospitals without morbidity [5,6]. In the US, over 98% of in-patients had morbidity defined by POMS [5] suggesting that patients with POMS score of 0 were rapidly discharged. In a previous UK study, 63% of orthopaedic patients remained in hospital with no morbidity on POD 3 and 42% on POD 5 suggesting that discharge efficiency was lower in the UK institution. In the US, many post-operative arthroplasty patients are discharged to a rehabilitation unit. This practice is far less common in the UK and may partially account for the discrepancy in discharge efficiency.

Use of POMS as a bed utilisation tool relies on the assumption that it captures all reasons for remaining in hospital. In this study, the main reason for remaining in hospital with no identifiable morbidity was ‘ongoing physiotherapy and occupational therapy input’. A specific concern in this patient group, who are often elderly and frail, is that they may not have adequate mobility to be discharged safely. Including a specific domain for mobility may improve the sensitivity of POMS for morbidity requiring hospitalisation following orthopaedic surgery. The domain should assess the ability to walk short distances, ability to climb a flight of stairs and a balance and falls assessment. Whilst this domain could be especially relevant for orthopaedic patients, this requires further investigation.

Use of POMS as a ‘fitness for discharge’ tool rests on the assumption that patients do not develop new morbidity after they have become free from morbidity, either in hospital or following discharge. No patients were readmitted to the study hospital in the first post-operative year for complications linked to surgery. However, 33 patients (6.2%) developed ‘new’ morbidity following a period without morbidity whilst in hospital. This highlights a limitation of prospectively using POMS as a ‘fitness for discharge’ tool. 25 of 33 (76%) of these patients developed wound morbidity after a period with no morbidity. To overcome this potential problem, primary arthroplasty patients should have regular wound reviews until POD 5 and revision arthroplasty patients until POD 8. This could be performed in an outpatient or primary care setting by a doctor or nurse. Extra capacity would be needed to ensure no negative impact on existing services. This would add cost to each patient episode but would be more cost effective than patients remaining in hospital. Patients should be aware of the risk of deep vein thrombosis, pulmonary embolus, myocardial infarction and cerebro-vascular accident following discharge and receive clear written instructions regarding symptoms and management. As long as these precautionary measures are in place, POMS has potential as a bed utilisation tool.

The most commonly used tool to assess appropriate bed utilisation in the literature is the Appropriateness Evaluation Protocol (AEP) [10]. AEP is a retrospective tool that relies on data from the inpatient record. It has been shown to be valid and reliable in some studies [10] but not in others [11]. POMS is a prospective tool that could be used in real time to assist with appropriate patient discharge. AEP is a retrospective tool that can only be used to evaluate past events. Data for POMS is collected directly from contemporary data sources whilst the patient is in hospital; AEP relies solely on past patient records and is therefore dependent on completeness and accuracy of record keeping for reliable functioning.

AEP has been used in several European countries to examine bed utilisation. In Portugal, 50% of inpatient days were deemed inappropriate [12], in Italy 37.3% [3], in Germany 28% [13], in Switzerland 8% to 15% [14] and in France 7% [15]. This study indicates bed utilisation in the UK is comparable to that seen in Portugal and Italy but such a direct comparison may have limited validity since different bed utilisation tools have been used.

The finding that many fewer patients remain in the hospital with no morbidity (as defined by POMS) in the US when compared with the UK suggests that bed utilisation in the US is superior to that seen in the UK. The implementation of ‘payment by results’ in the UK aims to improve appropriate bed occupancy to optimise patient care and improve efficiency. If the patients in this study had been discharged when they first had no morbidity defined by POMS, a saving of over £750,000 could have been made in 1 year (based on a cost of £400 per bed-day). This money could be reinvested in rehabilitation services with a strong emphasis on therapies rather than being spent on unnecessary inpatient medical care.

## Conclusions

We believe that POMS is able to identify patients remaining in hospital without clinically significant morbidity and may be used prospectively as a bed utilisation tool. To use the survey for this purpose, it may be useful to add an additional measure to assess mobility.

## Abbreviations

AEP: appropriateness evaluation protocol
ASA: American Society of Anesthesiologists
HR: hip resurfacing
POD: post-operative day
POMS: post-operative morbidity survey
THR: total hip replacement
TKR: total knee replacement
UK: United Kingdom
UKR: unicondylar knee replacement
US: United States (of America)
